# Measurement of perineal tears as an additional tool for laceration assessment during vaginal birth

**DOI:** 10.18332/ejm/174310

**Published:** 2023-12-20

**Authors:** Maristela B. M. Urasaki, Marlise O. P. Lima, Roselane Gonçalves, Natalucia M. Araújo, Carolina G. S. Pereira

**Affiliations:** 1 Midwifery Program, School of Arts, Science and Humanities, Sao Paulo University, Sao Paulo, Brazil; 2 State Hospital Carlos Lacaz, Sao Paulo, Brazil

**Keywords:** parturition, midwifery, nursing midwives, perineum, trauma, birth lacerations

## Abstract

**INTRODUCTION:**

Spontaneous lacerations at vaginal birth are everyday events, but their classification and management still challenge midwifery care. This study aims to measure and describe first-degree and second-degree perineal lacerations resulting from vaginal birth, describe their repair, and the education provided for care.

**METHODS:**

A descriptive study was conducted in a public maternity hospital in São Paulo, Brazil, with 87 parturients. Data were collected between October 2017 and June 2018 using a structured instrument containing obstetric variables and a description of lacerations. The obstetricians and nurse midwives assisted with births, determining the degree of laceration and intervention, and the researchers measured and reported them.

**RESULTS:**

The majority of parturients (82.7%) had lacerations only in the anterior region, 8% had them in the posterior region, and 9.2% in both regions. The lacerations were classified as first-degree (78.1%) or second-degree (21.8%). Among the 32 nulliparous parturients, 27.6% had first-degree lacerations, and 9.2% had second-degree. Of the 55 multiparous parturients, 50.6% had first-degree, and 12.6% had second-degree. Among the lacerations assessed as first-degree, 25% had deeper tissue layers compromised in addition to the skin and mucosa. There were 180 lacerations, with an average length of 33.1 mm, depth of 19.8 mm, and width of 23.8 mm. Half of the parturients did not receive guidance on laceration care. There was no association between parity and size, number, location, or degree classification of lacerations.

**CONCLUSIONS:**

This study provides a broad description of the characteristics of perineal lacerations and presents measurement techniques as a complementary resource for evaluating lacerations.

## INTRODUCTION

Perineal laceration resulting from vaginal birth can be defined as the loss of tissue integrity of the labia, vagina, urethra, clitoris, perineal muscles, anal sphincter, and rectum muscles, which can occur due to spontaneous tears or episiotomy. They are classified as first-degree, when the laceration affects only the skin and vaginal mucosa; second-degree, when it affects the perineal muscles but without compromising the anal sphincter; third-degree, when there is damage to the sphincteric complex; and fourth-degree, when there is damage to the external and internal sphincter complex and damage to the rectal mucosa^1^.

More than 85% of women who undergo a vaginal birth will suffer from perineal tears. These events occur globally and within different models of obstetric care^1,2^.

Two studies were conducted in teaching maternity hospitals in Brazil. The first included 222 parturients and identified 47% first-degree lacerations, 31% second-degree, and 1.8% third-degree^3^. The second study, with 226 primiparous women, recorded 32.7% of first-degree lacerations, 32.7% second, and 4.9% third^4^.

Perineal laceration from childbirth represents a significant cause of maternal morbidity because it results in several short- and long-term consequences for women’s health, such as pain and the potential for bleeding, infection, intestinal and urinary disorders, damage to the pelvic floor, and sexual alterations^5-7^. Third-degree and fourth-degree lacerations have a low incidence and are associated with more significant impairments such as genital prolapse, chronic perineal pain, and rectovaginal fistula^5,8^.

The incidence rate of 0.1–23.6% of infection in the lacerations was identified in a review study, despite clinical heterogeneity between investigations^9^. A study showed that the percentage of women with dyspareunia was 25%, 38%, and 53%, in women without laceration/first-degree, second-degree, and third/fourth-degree, respectively^10^. All these conditions have significant consequences, especially during motherhood^5-7^.

Spontaneous tears are everyday events, but their classification and management still represent a challenge in obstetric care^7,11^. The classification of tears is a complex task, considering the non-planar surface on which the laceration is located and other factors. The condition of the genitalia itself may favor or complicate the accuracy of the assessment, such as individual variations in the thickness of the adipose and muscle tissue in the vulvar and perineal regions. The amount of uterine bleeding and the extent of oedema and tearing may also make inspection difficult. The clinician’s ability and experience in identifying and describing the affected areas may also affect the accuracy of the perineal assessment^11^.

It has been reported that nurse midwives did not consistently classify perineal outcomes in vaginal births^12^. Variations above or below the degree of tear may be due to the lack of defined service protocols and the absence of standardized instruments for an objective evaluation. Another complication is the parturient degree of discomfort during the vulvar examination to classify tears.

Reliable assessments are fundamental for decision-making in conducting tissue recovery and minimizing short- and long-term adverse events^7-13,14^. Assessment tools that include laceration measurements have been helpful as they provide more accurate analyses than simple inspection. The measurement forms vary from the simplest, such as using measuring tapes, to the most complex with computer systems^15,16^. Monitoring laceration size has been seen as an adequate predictor to assess interventions’ healing potential and effectiveness^17^. Lacerations measurement has been frequently used in clinical and surgical practice as part of patients’ initial and follow-up assessments. However, it is not a frequent practice in obstetrics, as gathered from the available scientific data. Accurate lacerations measurement and description of types of tearing contribute to the proper allocation of human and material resources, estimation of procedure duration, the prognosis of the healing process, and risk identification, bearing in mind that these aspects will vary according to the severity and characteristics of lacerations.

The objectives of this study were to measure and describe first- and second-degree perineal lacerations resulting from vaginal birth, describe their repair, and the education provided on lacerations care.

## METHODS

### Study design, setting, and participants

A descriptive study was conducted in a public maternity hospital in the southern region of São Paulo City, Brazil. The population consisted of low-risk parturients assisted at the labor and birth ward, with no previous indication for cesarean birth. Convenience sampling was utilized. The inclusion criteria were full-term pregnancy, single gestation, cephalic presentation, and vaginal birth assisted by nurse midwives or obstetricians, with the occurrence of intrapartum perineal lacerations. The exclusion criterion was the occurrence of lacerations involving the anal sphincter or rectal mucosa (third- and fourth-degree lacerations, respectively), as they are more severe and were exclusively sutured by physicians in the current scope of practice.

### Study recruitment and ethical considerations

The parturients were invited to participate in the study right after admission to the labor and birth ward. Data were collected between October 2017 and June 2018.

The Committee for Ethics in Research approved this study. All ethical aspects (informed consent, confidentiality, data privacy, and instructions) related to research with human beings were respected. The permission of the study participants was granted by signing the Free and Informed Consent Term (TCLE) and Term of Free and Clarified Assent (for participants aged 12–18 years).

### Study variables and procedures for collecting the data

For data collection, a structured form was developed by the researchers that included sociodemographic data, clinical and obstetric variables (parity, history of vulvar injuries, and scars), characteristics of lacerations (site, number of lacerations, length, width, depth, presence of bleeding, oedema, and hematoma), laceration classification, and adopted management by the obstetricians and nurse midwives. To identify the laceration site, the following regions were considered: the anterior perineal triangle (periurethral, clitoris, middle vestibule, furcula, and vaginal walls) and the posterior perineal triangle (mid-lateral and median regions)^2^.

Sociodemographic data were extracted from medical records; the laceration classification was assigned by the obstetrician and nurse midwives who attended the childbirth and decided on the type of procedures implemented. Data relating to the guidance provided by the obstetrician and nurse midwife on postpartum laceration care were collected directly from the parturient before being transferred from the labor and birth ward to the rooming-in with their baby.

The obstetricians and nurse midwives, part of the hospital staff, assisted the parturient during labor and birth. They were also responsible for assessing and repairing any perineal lacerations during the process. All had been educated on assessing and repairing vaginal birth lacerations during their respective undergraduate courses. The births occurred in different positions, but the assessments were performed with the parturient in the gynecological or lithotomy position.

The research team consisted of three experienced nurse midwives and one dermatology nurse (all of whom were instructors in a midwifery course at a public university in São Paulo), and one midwifery student. The hospital of this study served as a clinical setting for midwifery students.

The researchers were responsible for measuring lacerations and recording the findings without giving an opinion regarding the classification of the tears or their management. Subsequently, what the obstetricians and nurse midwives described was verified and compared with the measurements obtained.

To standardize the method of measuring and describing the tears, all researchers participated in the four training meetings preceding the events. Three researchers were responsible for performing the measurements. At these meetings, different formats and dimensions of tears were projected using photos, drawings, and painted foam molds to determine the manner of measurement. It was defined as follows: 1) the skin and local structures would be pulled only enough to visualize the affected area, 2) tears with irregular edges would be measured at their greatest lengths and widths, 3) V tears would be measured using the lengths of the vertices towards the superior centers, and 4) the depth would also be measured at the place of its largest measurement. The first five measurements and characterizations were pilot tests to standardize the measurement technique. After performing the five collections, adjustments were not required.

The measurement procedure was performed after birth (<30 minutes) with a simple and low-cost method using sterile, flexible, disposable oxygen catheters. One researcher opened the package and placed it over the laceration, initially checking the length. The portion of the measured catheter was cut, and if there were distant edges and deepening of the laceration, the width and depth with the catheter. After each cut, the catheter piece was given to another researcher, who measured its size. The procedure was performed multiple times to measure all lacerations visualized. The assistant investigator simultaneously recorded all pertinent information in the collection instrument. For the tear measurements, the cutting catheters were matched to the numerical scale of a standard ruler, and the corresponding values in mm were recorded.

The type of measurement performed was based on the damaged tissue. Superficial tears and those with approximated edges could only be measured in length. Superficial tears with far edges were measured in length and width between the walls. The non-superficial tears allowed the introduction of the catheters in the inferior layers to measure the depth, wherein the length was also verified. When possible, width measurement was also performed, hence the three measures. In measuring the lengths and widths, the catheter was brought close to the damaged tissue without touching it; for depth measurement, the catheter was carefully and lightly overlapped with the injured tissue to avoid further discomfort to the woman.

The following parameters were used to describe oedema and bleeding: absent, little/small, moderate, and abundant/large. For the quantification of oedema, the oedema item in the Redness, Oedema, Ecchymosis, Discharge, Approximation (REEDA) scale was used as a basis^18^ in which minor oedema was equivalent to <1 cm from the laceration, moderate of 1–2 cm from the laceration, and >2 cm. The number of saturated gauzes was considered to quantify bleeding, and five saturated gauzes were equivalent to approximately seven mL of blood. The criterion adopted for minor bleeding was up to five saturated gauzes, average from six to ten and abundant more than eleven gauzes. For hematoma, it was considered present or absent.

### Statistical analysis

The collected data were coded using Microsoft Excel^TM^. Descriptive statistical analysis was performed on absolute and relative numbers for categorical variables and measures of central tendency and dispersion for quantitative and inferential variables using the chi-squared and Kruskal-Wallis tests. The significance level adopted was 5% (p<0.05), and the Minitab^TM^ version 18 program was used for the analyses.

## RESULTS

The study consisted of 87 parturients aged 16–41 years (mean: 25.8 ± 7.24), 37.9% of whom were aged 19–22 years. The shortest formal schooling time was five years, and 53.9% had studied to high school level. Regarding parity, 55 (63.2%) had had one or more births. No parturient received epidural anesthesia.

Regarding the clinical history of the vulvar and anal region, 11 (12.6%) parturients reported hemorrhoids, 6 (6.8%) had vulvar varices, one reported scarring due to bartholinitis, and 35 (40.2%) had episiotomy scar(s) from previous births.

A total of 180 tears were identified and measured in different locations of the vulvar region. It was found that 72 (82.7%) parturients had tears only in the anterior region, 7 (8%) in the posterior region, and 8 (9.2%) in both regions (Table 1).

**Table 1:** Number of perineal tears and location in the vulvar region, among study participants, São Paulo, 2017–2018 (N=87)

*Variable*	*n*	*%*
**Number of tears (n=87)**		
1	37	42.5
2	19	21.8
3	21	24.1
4	8	9.1
6	2	2.2
**Location (n=180)**		
Furcula	47	26.1
Right periurethral	36	20.0
Left periurethral	31	17.2
Right middle vestibule	22	12.2
Left middle vestibule	17	9.4
Right lateral medium	10	5.5
Clitoris	7	3.8
Middle left side	6	3.3
Vaginal wall	2	1.1
Median	2	1.1

The length was measured for all tears, while 39 (21.6%) and 46 (25.6%) had their width and depth measured, respectively. The mean length was 33.1 ± 17.3 mm, the width 23.8 ± 11.8 mm, and the depth 19.8 ± 10.4 mm. Of all the participants, length alone was measured in 116 (64.4%) parturients, length and depth in 25 (13.8%), length and width in 18 (10%), and all three dimensions in 21 (11.6%) parturients.

Sixty-eight (78.1%) parturients had spontaneous first-degree tears, and 19 (21.8%) had second-degree tears. Among the 32 nulliparous, 24 (27.6%) had first-degree tears, and eight (9.2%) had second-degree tears. Of the 55 multiparous parturients, 44 (50.6%) had first-degree tears, and 11 (12.6%) had second-degree tears.

In the 68 parturients with first-degree tears, there were a total of 136 lacerations; 95 (69.8%) could only be measured in length, 16 (11.7%) in length and depth, 14 (10.2%) in length and width, and 11 (8%) in all three dimensions. Length measurements ranged from 6.0–95.0 mm; width, 5.0–39.0 mm; and depth, 6.0–38.0 mm. In this group, 26 (38.2%) parturients had tears measured in depth (one with two tears) and 17(25%) with a depth greater than 11 mm.

In the 19 parturients with second-degree tears, there were a total of 44 lacerations, 21 (47.7%) could only be measured in length, 9 (20.4%) in length and depth, 4 (9.0%) in length and width, and 10 (22.7%) in length, width, and depth. Length measurements ranged from 10.0–76.0 mm; width, 17.0–63.0 mm; and depth, 6.0–43.0 mm. Table 2 shows the distribution of lacerations’ length, width, depth measurements, and the degree of laceration.

**Table 2:** First-degree and second-degree tears’ length, width, and depth, among study participants, São Paulo, 2017–2018 (N=180)

*Measurement range (mm)*	*1st degree*						*2nd degree*					
	*Length*		*Width*		*Depth*		*Length*		*Width*		*Depth*	
	*n*	*%*	*n*	*%*	*n*	*%*	*n*	*%*	*n*	*%*	*n*	*%*
5–10	6	4.5	3	12.0	9	33.3	1	23.0			1	5.2
11–20	33	24.3	12	48.0	12	44.5	6	13.6	4	28.6	7	36.9
21–30	48	35.3	6	24.0	4	14.8	9	20.4	6	43.0	4	21.0
31–40	21	15.4	4	16.0	2	7.4	9	20.4	1	7.1	6	31.7
41–50	14	10.3					7	16.0	1	7.1	1	5.2
51–60	7	5.2					7	16.0	1	7.1		
61–70	1	0.7					4	9.0	1	7.1		
71–80	3	2.2					1	2.3				
81–90	2	1.4										
90–95	1	0.7										
Total	136	100	25	100	27	100	44	100	14	100	19	100

Regarding the amount of bleeding in the injured area, most parturients had minor bleeding; it was considered absent in five (5.7%), minor in 43 (49.4%), moderate in 34 (39%), and abundant in five (5.7%) parturients. Few parturients had other local tissue modifications; oedema was present in eight (9.1%) parturients, three (3.4%) of which were moderate (10–20 mm) and five (5.7%) were insignificant (<10 mm). Four parturients (4.5%) developed a local hematoma.

Table 3 lists the findings of the suture procedure performed by the obstetricians and nurse midwives.

**Table 3:** Bleeding control, suture, type of suture, and threads used, among study participants, São Paulo, 2017–2018 (N=87)

*Variable*	*n*	*%*
**Compressive bleeding control**	63	72.4
**Suture (n=87)**		
1st-degree tears	36	41.3
2nd-degree tears	19	21.8
**Type of suture (n=55)**		
Continuous in all plans	29	52.7
Continuous in skin/mucosa	14	25.5
Separated single dots	9	16.4
Continuous modified*	3	5.4
**Threads used in the suture (n=55)**		
Simple catgut	36	65.4
Polyglycolic 910	19	34.5

* Continuous suture in mucosa with closure in this layer. Then continuous in muscle and skin with closure in these layers.

Of the 36 (41.3%) parturients sutured with first-degree tears, 69 lacerations occurred in 16 (18.3%), the tears were superficial, and the depths were not measured; in 12 (13.7%) participants, the tears had depths greater than 11 mm, in 8 (9.1%) they were less than 10 mm. In this group’s total, the smallest depth measurement was 5 mm, and the largest was 38 mm. However, six (6.8%) participants with first-degree lacerations that were not sutured had a measured depth greater than 11 mm. Among the 19 (34.5%) parturients with tears sutured and classified as second-degree, one measured depth was 6 mm.

Regarding instructions provided to parturients in the labor and birth ward, 50.5% did not receive instructions regarding laceration care. Aspects related to vulvar hygiene were the most discussed, which means washing with soap and water in a bath (42.5%), washing with water after urination (25.2%), using moistened tissue after urination or defecation (2.3 %), keeping the region dry (2.3%), and using warm water for local hygiene (2.3%). Other recommendations included explaining the stitches’ absorption (13.8%), burning of the vulva during first vesical elimination (8%), use and change of pads (4.6%), and sexual abstinence for 40 days (1.1%).

The inferential analysis using the Kruskal-Wallis test did not reveal any significant difference between nulliparous and multiparous parturients in terms of laceration length (DD=1; H-value=0.13; p=0.718), width (DD=1; H-value=1.38; p=0.240), and depth (DD=1; H-value=0.67; p=0.414). No association was found using the chi-squared test between parity and classification of laceration degree (p=0.586) or with the number of lacerations (p=0.286) or perineal region (p=0.944).

## DISCUSSION

Accurately identifying and describing perineal tears requires professional skills, as several factors make it challenging to implement visual inspection. Differences in women’s skin characteristics and local structures are complicating elements; the few studies of normal and intact external female genitalia show variations in their measurements^19^.

Researchers who analyzed the size, symmetry, and morphology of vulvar structures in adolescents found significant variation^20^. The presence of genetic alterations and scars also makes the examination complex. As a result, the inspection and assessment of the vulva and perineum, associated with the event of vaginal birth, becomes even more difficult because it adds exclusive aspects to this condition, such as oedema, bleeding, ecchymosis, and pain^11^.

In this study, a significant number of parturients were declared to have episiotomy scars, and a small number reported other vulvar alterations before pregnancy. Skin containing scarring does not have the same tissue architecture as the original skin, which may impact tissue regeneration. In addition, episiotomy scars are associated with perineal tears in subsequent childbirth^21^.

Regarding the site of tears, there is no consensus in the literature for the delimitation/description of perineal areas, making comparing with other studies complex^22^. The findings of this investigation are compatible with those presented in a peri-hospital birth center in São Paulo, wherein 63.8% of tears occurred in the right and left labial, vestibule, and anterior vaginal wall, and 36.2% in the perineal body and posterior vaginal wall. Other researchers found that lacerations were more common in the posterior region (52%)^22^.

Most parturients in this study had more than one tear, and few researchers have reported and analyzed this phenomenon. Multiple lacerations can compromise tissue structures and layers, leading to more significant morbidity. Less tissue loss is associated with a higher healing rate, requiring less tissue repair^23^. Larger tears require more prolonged healing, and this can increase infection risk^24^.

Tear measurements varied in length, width, and depth, which could lead to different results. A clinical trial that measured the depth of lacerations as a parameter to assess the effectiveness of the applied intervention found a mean depth of 2nd-degree lacerations of 23 mm in the control group^25^. Monitoring the size of lacerations is a valuable tool for evaluating lacerations progression, so it is essential to have data from the first measurement^26^.

In our investigation, perineal lacerations with greater lengths (>51 mm) represented the minority in this study, did not have depth, and only two had measured width. The lacerations with shorter lengths (<40 mm), which accounted for the majority, were tears that allowed for measuring both width and depth. These findings indicate that many women suffered significant tissue lacerations in a limited perineal area, and more extensive lacerations were associated with a lower healing rate^23^. Although dealing with lacerations of a different nature, this association emphasizes the importance of rigor in monitoring the healing process of tissue laceration. Observational, prospective research that analyzed electronic medical records of 828 women, tracked infections in second-degree lacerations and found 16 (1.9%) laceration-related infectious conditions^27^.

Most parturients in this study had first-degree tears, as classified by an obstetrician or nurse midwife, consistent with other studies^3,22,28^. However, measurements of laceration depths greater than the epidermis and dermis thickness, were found. The study of the human skin has established that the epidermis presents topographical variations of 0.04–1.60 mm, and the dermis from 1–4 mm, up to 5.6 mm^29^. The skin of the vulva measured by ultrasonography showed that the mean thickness of the epidermis of the labia majora was 0.21 mm, with the greatest value of 3.4 mm. It showed the dermis of the labia majora mean of 2.21 mm and greatest value of 4.32 mm; the epidermis of the labia minora mean of 0.08 mm and greatest value of 0.32 mm; the dermis of the labia minora mean of 1.93 mm and greatest value of 5.49 mm^30^. The established maximum measurements of the epidermis and dermis, whether for the labia majora or minora, are less than 7.8 mm. In this investigation, 17 parturients had lacerations measured deeper than 10 mm, 11 of which were greater than 16 mm, classified as first-degree lacerations. It was observed that the obstetricians and nurse midwives in this study tended to underestimate the degree of laceration.

In this sense, depth measurement can be a complementary resource to determine the degree of the tear and avoid evaluation errors, considering that the measurement value is objective.

The decision on the suture procedure was variable among obstetricians and nurse midwives for lacerations classified as first-degree. Some superficial lacerations were sutured without depth measurements or depths less than 10 mm. The researchers understand that this conduct may have been adopted for hemostatic or aesthetic control. Given the possible muscle involvement, parturients with tears larger than 11mm had their lacerations sutured. However, six parturients with deep and bleeding lacerations were not sutured. The reasons for these are unclear.

Another issue related to suturing was the variation in the selected technique and type of thread. There was no protocol for this procedure in the hospital where the study was conducted. However, robust evidence supports continuous suturing in all tissue layers and indicates the most appropriate thread^1^.

In this investigation, a few cases of hematomas were identified. A study that found a higher frequency of hematomas (39% in primiparas and 11% in multiparous parturients) evaluated this condition using ultrasound, not visual inspection^31^. Oedema, an important finding in the perineal assessment and promoting local pain, was uncommon in this study.

Several researchers have reported the relationship between spontaneous tear and parity, and nulliparity has been considered a risk factor^28,32^. In this study, there was no association between tear size, degree, number, and location with parity, although second-degree tears were more frequent among nulliparous parturients. These findings have not been documented previously and should be confirmed by additional studies.

Regarding the advice that obstetricians and nurse midwives provided on postpartum perineal care, there was diversity in the parturients’ responses, an expected fact considering that the service did not have a guidance protocol. Although perineal care instructions are crucial to promoting a good healing process, no investigations analyzing the guidelines for this population were found in the reviewed literature.

The findings of this investigation corroborated the importance of using instruments that are easy to apply clinically for the measurement of lacerations and the implementation of protocols by obstetric/midwifery care services^11,15,16,33^. Besides being valuable for monitoring the healing process, laceration measurement as an assessment tool also helps in the learning processes^34^. In addition, measurement is precious for novice obstetricians, midwives, and nurses midwives, who are still acquiring and improving their skills and need support for developing clinical competence and confidence in choosing the best approach for tissue recovery, such as suturing or not.

### Limitations

The limitations of this study are found in the use of a non-validated measurement tool developed by the researchers for this investigation, low sample size, and the convenience type, conditions that make it impossible to generalize the results. Further studies should apply standardized procedures with standardized tools for assessing spontaneous lacerations. However, this study provides an interesting insight into this study population.

## CONCLUSIONS

The participants in this study had multiple lacerations in the anterior part of the perineum. Regarding length, the average was 33.1 mm, depth 19.8 mm, and width 23.8 mm; oedema and hematoma were uncommon events, as well as profuse bleeding. This study’s clinicians who assisted the vaginal births classified 78.1% as first-degree lacerations. However, these measurements suggest that deeper tissue layers were impaired, not only the skin and mucosa.

No association was noted between parity and tear size, grade, number of traumas, and location.

Measuring the dimensions of perineal lacerations is a complementary resource for evaluating perineal lacerations associated with childbirth.

## Data Availability

The data supporting this research are available from the authors on reasonable request. Further details can be found in the Supplementary file.
