# Supplementary material for: Measurement of perineal tears as an additional tool for laceration assessment during vaginal birth
Source: Eur J Midwifery. 2023 Dec 20;7:43. doi: 10.18332/ejm/174310 (PMC10731751; doi:10.18332/ejm/174310)
Supplement: Supplementary file 1 [file EJM-7-43-s1.pdf]

Data collection instrument

Name __________________________ Form Nr. ___________
Date and time of hospitalization_______________ Medical record nr _________

General data

Age _____________ Schooling _____________________ Occupation___
Skin color: yellow ○ caucasian ○ black ○ brown ○

Skin and perineum health history

Previous episiotomy	no ○	yes ○	Nr:____________
Other scars	no ○	yes ○	Cause: _____________
Vulvar varices	no ○	yes ○	
Hemorrhoids	no ○	yes ○	
Local infections	no ○	yes ○	

Obstetric History

Gestation nr: _______ Parity ______Abortions ______ Number of children _________

Professional who performed the delivery:

NM ○ ObGyn ○ Resident ○ Midwife○

[NM: nurse midwife; ObGyn: obstetrician gynecologist; Resident: Medical Resident]

Tear assessment

Total number of tears ___________________

Location of tear 1 ___________________

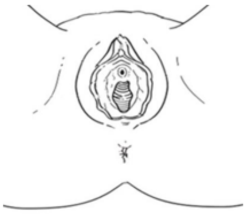

Length ___________
Width ___________
Depth ___________

Location of tear 2 __________________

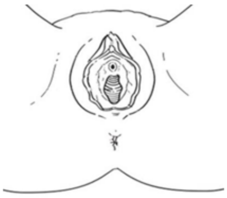

Length __________
Width __________
Depth __________

Location of tear 3 __________________

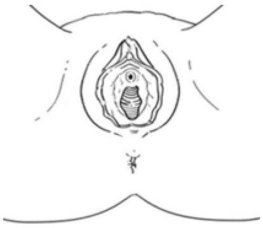

Length ___________
Width ___________
Depth ___________

Location of tear 4 ___________________

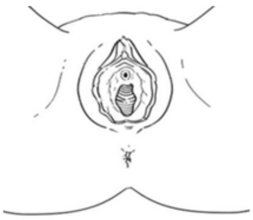

Length ___________
Width ___________
Depth ___________

Location of tear 5 _________________

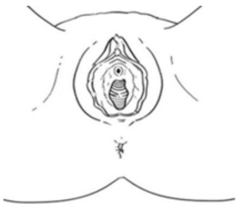

Length ___________
Width ___________
Depth ___________

Location of tear 6 _______________

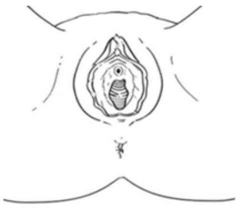

Length ___________
Width ___________
Depth ___________

General aspect

Bleeding: absent ○ light ○ moderate ○ excessive ○

Edema (adapted REEDA scale):

(-) absent ○ (+) small [less than 1 cm from the incision ○ (++) moderate [perineal or vulvar 1-2 cm from the incision] ○ (+++) large [perineal or vulvar beyond 2 cm of the incision] ○

Hematoma no ○ yes ○

Tear classification (professional assessment)

1st grade ○ 2nd grade ○

Post trauma immediate procedures

Bleeding control by compression no ○ yes ○

Suture no ○ yes ○

Thread used _________________ Surgical glue ______________

Type of suture: total continuous ○ continuous in the mucosa; muscle and skin separate stitches ○ modified continuous (only separate stitches on muscle) ○

Guidelines on postpartum tear care offered by the professional (describe)

Researcher name______________________________________

© 2023 Urasaki M. B. M. et al.
